# Association between Corneal Stiffness Parameter at the First Applanation and Keratoconus Severity

**DOI:** 10.1155/2020/6667507

**Published:** 2020-12-02

**Authors:** Kaili Yang, Liyan Xu, Qi Fan, Shengwei Ren

**Affiliations:** Henan Provincial People's Hospital, Henan Eye Hospital, Henan Eye Institute, People's Hospital of Zhengzhou University, Henan University People's Hospital, Zhengzhou 450003, China

## Abstract

**Objective:**

The study aimed to evaluate the character of corneal stiffness parameter at the first applanation (SP-A1) in normal and keratoconus eyes and explore the association between SP-A1 and keratoconus severity indicators.

**Methods:**

A total of 351 normal and 351 keratoconus eyes were included in the current study. Keratoconus was diagnosed according to the corneal topography map and slit-lamp examination. The severity of keratoconus was classified to mild (steep keratometry (Ks) < 48D), moderate (48 ≤ Ks < 55D), and severe (Ks ≥ 55D). The SP-A1 was measured using the Corvis ST software. The correlation analyses and receiver operating characteristic (ROC) curve were performed in the current analysis.

**Results:**

The SP-A1 values of keratoconus were lower than that of normal eyes (72.11 (57.02, 83.08) mmHg/mm vs 110.89 (100.45, 122.47) mmHg/mm, *P* < 0.001). With the severity of keratoconus increasing, the SP-A1 decreased and the value of SP-A1 was 79.54 (70.30, 90.93) mmHg/mm, 65.11 (53.14, 77.46) mmHg/mm, and 47.59 (37.50, 62.14) mmHg/mm in mild, moderate, and severe keratoconus eyes, respectively (*P* < 0.001). The negative association between SP-A1 and Ks was found in mild, moderate, and severe keratoconus eyes (r _mild_ = -0.171, *r*_moderate_ = -0.317, *r*_severe_ = −0.288, all *P* < 0.05). A positive association between SP-A1 and the thinnest corneal thickness (TCT) was found in all eyes (r_normal_ = 0.687, *r*_mild_ = 0.519, *r*_moderate_ = 0.488, *r*_severe_ = 0.382, all *P* < 0.05). SP-A1 was found to be statistically positively associated with intraocular pressure (IOP), biomechanical corrected IOP (bIOP), time from the initiation of air puff until the first applanation (A1T), corneal velocity at the second applanation (A2V), and negatively associated with deformation amplitude (DA), peak distance (PD), corneal velocity at the first applanation (A1V), time from the initiation of air puff until the second applanation (A2T), and DA Ratio Max [2 mm] both in normal and keratoconus eyes (all *P* < 0.05). The ROC analysis indicated that the AUC (95% CI) of SP-A1 was 0.952 (0.934–0.967) and 0.930 (0.904–0.951) in detecting keratoconus eyes and mild keratoconus eyes from normal eyes, respectively.

**Conclusions:**

The SP-A1 value decreased while the keratoconus severity increased. It was lower in keratoconus than that in normal eyes and could be helpful in identifying keratoconus eyes from normal eyes. Further researches would be warranted to expand the clinical utility of SP-A1.

## 1. Background

Keratoconus is an asymmetrical bilateral corneal ectasia that causes significant visual morbidity [1]. The corneal tomographic or topographic maps could help to diagnose keratoconus in the alteration of the corneal shape [2]. Previous studies have found that abnormal corneal biomechanics, which could be detected by an ophthalmology device, might occur before the changes of corneal tomographic maps [3, 4]. In addition, corneal cross-linking (CXL) is a photochemical reaction utilizing ultraviolet (UV) A light and riboflavin as a photosensitizer, and is safe and effective for keratoconus patients [5–7]. Recent studies have reported that CXL could halt the progress of keratoconus patients by increasing the corneal stiffness [8, 9]. Thus, the assessment of corneal stiffness in keratoconus eyes has been recently gaining more attention with an increase in the popularity of CXL.

Stiffness parameter at the first applanation (SP-A1) is a novel parameter in the form of force divided by displacement at the first applanation, which could be measured by Corneal Visualization Scheimpflug Technology (Corvis ST) [3, 10]. Several studies have reported that the values of SP-A1 in normal eyes ranged from 89.32 mmHg/mm to 148.95 mmHg/mm [11, 12], which were higher than that of keratoconus eyes that ranged from 46.6 mmHg/mm to 77.16 mmHg/mm [13, 14]. The discrepancy of corneal biomechanical parameter might explain the etiology of keratoconus [1]. The classification of keratoconus is of great significance for understanding the disease progression and choosing a reasonable treatment for a patient [1, 15]. Therefore, it is meaningful to evaluate the association between keratoconus severity indicators and SP-A1 values in clinical application. A recent study found that SP-A1 was positively associated with the thinnest corneal thickness (TCT) in three different grades of keratoconus, while the relationship with steep keratometry (Ks), which is an important classification indicator, was not reported [15]. In addition, several studies found SP-A1 was related to intraocular pressure (IOP) and other corneal biomechanical parameters in normal eyes; however, these relationships in keratoconus eyes were limited reported and not exactly consistent [3, 16]. Furthermore, several studies reported that the discriminative ability of SP-A1 for detecting keratoconus eyes from normal eyes were still existed differences [13, 17, 18].

Thus, the study aims to assess the values of SP-A1 in normal and keratoconus eyes, and further explore the asssociation between SP-A1 and keratoconus severity indicators. Furthermore, the discriminating ability of SP-A1 in identifying keratoconus eyes and mild keratoconus eyes from normal eyes was evaluated.

## 2. Methods

### 2.1. Study Subjects

Keratoconus was diagnosed according to the following criteria: an asymmetric bow tie pattern with or without skewed axes revealed by corneal topography map or keratoconus sign on slit-lamp examination, such as conical protrusion, localized stromal thinning, Fleischer's ring, Vogt's striae, or anterior stromal scar [1, 18]. Subjects scheduled for refractive surgery with spherical equivalent less than 8.00 diopters (D), corneal astigmatism less than 1.50 D, and the value of corrected distance visual acuity (CDVA) in LogMAR no more than 0.1 were included in the normal control group. Eyes with an anterior stromal scar, other eye diseases or any ocular surgery, rigid contact lens used in the last 4 weeks, soft contact lens used in the last 2 weeks, and serious diabetes were excluded. Finally, a total of 351 keratoconus eyes (248 patients) and 351 normal eyes (351 subjects) were recruited in the analyses. This study was approved by the Institutional Review Board of Henan Eye Hospital [ethical approval number: HNEECKY-2019 (5)], and written informed consent was signed by each participant.

### 2.2. Examinations

The basic characteristics of participants were collected through medical records. The slit-lamp examination and ophthalmoscope examination were performed by an experienced operator. The axial measurement (AL) and anterior chamber depth (ACD) were measured using the IOL Master. The mean endothelium cell density (MCD) was measured by a noncontact specular microscope. The corneal tomographic measurements were performed by Pentacam HR software, and Ks, flat keratometry (Kf), mean keratometry (Km), and TCT values were recorded. The severity of keratoconus was classified as mild (Ks < 48D), moderate (48 ≤ Ks < 55D), and severe (Ks ≥ 55D).

SP-A1 was measured through Corvis ST (Oculus, Wetzlar, Germany), which contains the first applanation, highest concavity, and the second applanation. SP-A1 is defined as resultant pressure, which is calculated as adjusted pressure at the first applanation (adj AP1) minus IOP, divided by deflection amplitude at the first applanation (A1DLA). The equation is as follows: SP-A1 = (adj AP1−bIOP)/A1DLA [3]. Among these parameters, the adj AP1 is regarded as the air pressure pumping on the cornea at the time and position of applanation, and the biomechanical corrected IOP (bIOP) is calculated through finite element simulations that take into account the influence of age, central corneal thickness (CCT), and other dynamic corneal response parameters [19]. In addition, IOP, deformation amplitude (DA), peak distance (PD), radius, time from the initiation of air puff to the applanation status (containing A1T, HCT, and A2T), and the corneal velocity and deflection length of the first applanation (A1V, A1DLL), second applanation (A2V, A2DLL), the maximum deformation (HCDLL), DA Ratio Max [2 mm], and DA Ratio Max [1 mm] were also measured in the current study. Measurements with good quality scores (QS, where the QS status is OK) that enabled calculation of corneal biomechanical parameters were included in the analyses.

### 2.3. Statistical Analysis

Data in normal, mild, moderate and severe keratoconus eye groups was not normally distributed, so median (interquartile range, IQR) was applied to describe the values of parameters. The SP-A1 in normal, mild and moderate keratoconus was compared by general linear model. The Spearman's rank correlation tests were used to investigate the relationship between SP-A1 and Ks, Kf, Km, TCT and other Corvis ST parameters. The receiver operating characteristic (ROC) curve was used to evaluate the diagnostic ability of SP-A1 for distinguishing keratoconus eyes and mild keratoconus eyes from normal eyes. The statistical analyses were performed using the SPSS 23.0 software package and MedCalc software, and a *P* < 0.05 (two-tailed) was considered statistically significant.

## 3. Results

### 3.1. Demographic Data of the Participants

Table 1 shows the clinical characters of normal eyes and keratoconus eyes. The ages between normal eyes (24.00 (19.00, 27.00) years) and keratoconus eyes (24.00 (20.00, 30.00) years) were found no statistically difference (*P* = 0.356). Compared to normal eyes, the values of ACD and Km in keratoconus eyes were higher and increased with the increase in the severity of the disease (all *P* < 0.05), and the values of IOP, AL, MCD, and TCT in keratoconus eyes were lower and decreased with the increase in the severity of the disease (all *P* < 0.05).

### 3.2. Distributions of SP-A1

**Figure 1:**
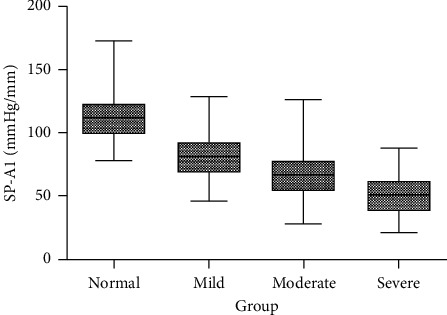
Distribution of SP-A1 in normal and keratoconus eyes.

The distributions of SP-A1 in normal and keratoconus eyes are presented in Figure 1. The SP-A1 values of keratoconus were lower than that of normal eyes ((72.11 (57.02, 83.08) mmHg/mm vs 110.89 (100.45, 122.47) mmHg/mm, *P* < 0.001). With the increase in the severity of the disease, the SP-A1 decreased and the values were 79.54 (70.30, 90.93) mmHg/mm, 65.11 (53.14, 77.46) mmHg/mm, and 47.59 (37.50, 62.14) mmHg/mm in mild, moderate, and severe keratoconus, respectively (*P* < 0.001).

### 3.3. Association between SP-A1 and Keratoconus Severity

**Figure 2:**
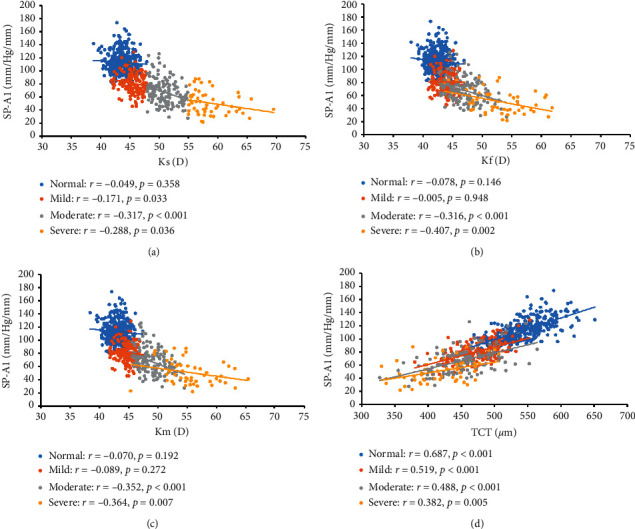
Association between SP-A1 and keratoconus severity. (a) Association between SP-A1 and Ks. (b) Association between SP-A1 and Kf. (c) Association between SP-A1 and Km. (d) Association between SP-A1 and TCT.

The association between SP-A1 and keratoconus severity indicators is shown in Figure 2. The negative association between SP-A1 and Ks was found in mild, moderate, and severe keratoconus eyes (*r*_mild_ = -0.171, *r*_moderate_ = −0.317, *r*_severe_ = −0.288, all *P* < 0.05), while the association in normal eyes was not statistically significant (Figure 2(a), *P*=0.358). SP-A1 was found to be negatively related to Kf and Km in moderate and severe keratoconus eyes (SP-A1 vs Kf: *r*_moderate_ = −0.316, *r*_severe_ = −0.407, all *P* < 0.05; SP-A1 vs Km: *r*_moderate_ = −0.352, *r*_severe_ = −0.364, all *P* < 0.05), while no statistically significant association was found in normal and mild keratoconus eyes (Figures 2(b) and 2(c), all *P* > 0.05). In addition, positive association between SP-A1 and TCT was found in all eyes (Figure 2(d), *r*_normal_ = 0.687, *r*_mild_ = 0.519, *r*_moderate_ = 0.488, *r*_severe_ = 0.382, all *P* < 0.05).

### 3.4. Association between SP-A1 and Corvis ST Parameters

The association between SP-A1 and Corvis ST parameters is shown in Table 2. SP-A1 was found to be statistically positively associated with IOP, bIOP, A1T, A2V, and negatively associated with DA, PD, A1V, A2T, and DA Ratio Max [2 mm], both in normal and keratoconus eyes (all *P* < 0.05). The statistically positive association between SP-A1 and radius (*r*_normal_ = 0.168, *r*_mild_ = 0.260, *r*_moderate_ = 0.391), and negative association between SP-A1 and DA Ratio Max [1 mm] (*r*_normal_ = −0.474, *r*_mild_ = −0.376, *r*_moderate_ = −0.509) was found in normal, mild, and moderate keratoconus eyes (all *P* < 0.05). Statistically negative association was found between SP-A1 and A1DLL (*r*_normal_ = −0.107, *r*_mild_ = −0.286), SP-A1 and HCDLL (*r*_normal_ = −0.474, *r*_mild_ = −0.376) in normal and mild keratoconus eyes (all *P* < 0.05). Statistically negative association between SP-A1 and A2DLL was found in moderate keratoconus eyes (*r*_moderate_ = −0.252, *P* = 0.003).

### 3.5. ROC Curve Analysis

**Figure 3:**
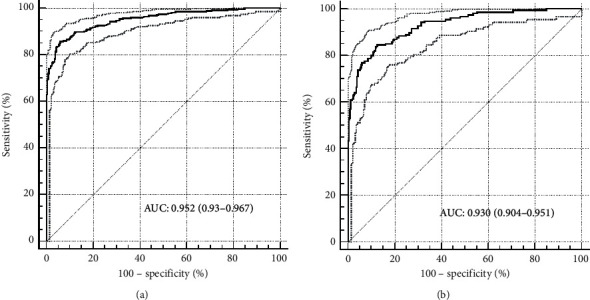
ROC analysis of SP-A1 for detecting keratoconus eyes and mild keratoconus eyes from normal eyes. (a) All keratoconus eyes. (b) Mild keratoconus eyes.

The ROC analysis results indicated that the area of ROC (AUC) (95% confidence interval (CI)) of SP-A1 was 0.952 (0.934–0.967) in detecting keratoconus eyes from normal eyes (Figure 3(a)). With a cutoff value of 91.64 mmHg/mm, 78.77% of keratoconus eyes were correctly classified with 85.75% sensitivity and 94.02% specificity. The AUC (95% CI) of SP-A1 was 0.930 (0.904–0.951) in detecting mild keratoconus eyes from normal eyes (Figure 3(b)). With a cutoff value of 95.75 mmHg/mm, 72.08% of mild keratoconus eyes were correctly classified with 84.62% sensitivity and 87.46% specificity.

## 4. Discussion

SP-A1 is an important biomechanical parameter that reflects the stiffness of cornea. The case control study found that the SP-A1 value in keratoconus eyes was lower than that in normal eyes. The SP-A1 decreased with an increase in the severity of keratoconus, and was positively associated with TCT, and negatively associated with Ks, Kf, and Km. In addition, the diagnostic analyses indicated that SP-A1 could help distinguish mild keratoconus eyes from normal eyes in clinical application.

The study showed that the SP-A1 in Chinese normal eyes was lower than previous results reported by Fernandez et al. [12], Sedaghat et al. [17], and Qin et al. [20], and higher than other studies shown in Supplementary Table 1 [3, 11, 13, 15, 16, 18, 21–32]. The SP-A1 value in keratoconus eyes of our study was lower than previous studies conducted by Sedaghat et al. [33], Kataria et al. [13], and Hashemi et al. [9], and higher than other studies shown in Supplementary Table 1 [3, 14, 15, 17, 18, 21, 22, 25, 32, 34, 35]. This discrepancy might be attributed to the study population diversity, sample size discrepancy, and the inconsistent inclusion criteria of the study subjects. Thus, a multicenter study should be conducted to explore the distribution of SP-A1 in different populations in the future. In addition, the current study found that the SP-A1 value in keratoconus eyes was lower than that in normal eyes, which was similar to other reports [3, 13, 17, 21]. The differences in SP-A1 values indicated that keratoconus eyes had weaker corneal stiffness than normal eyes, which provide reference for exploring the etiology of keratoconus.

Knowing the classification of keratoconus could help patients to choose a reasonable treatment. As an important parameter of the ocular optical system, the keratometry value stands for the optical refractive power of the eye and is an important keratoconus severity indicator [36]. Previous study has demonstrated that the SP-A1 was negatively correlated with the keratometry value in myopic subjects, which imply that the steep cornea usually had a lower corneal stiffness [32]. A recent study reported that the SP-A1 values were 74.85 ± 19.35 mmHg/mm, 60.43 ± 12.65 mmHg/mm, and 38.87 ± 17.66 mmHg/mm in Ks < 55 D, 55–62D, and ≥62 D keratoconus eyes, respectively, and the SP-A1 was negatively related with the keratometry value in the Ks < 55 D group [16]. The current study also found that the SP-A1 values in keratoconus eyes decreased with an increase in the Ks, which further indicated that SP-A1 could be a valuable clinical parameter that enables to biomechanically track the progression of keratoconus [16]. In addition, the study found that SP-A1 was negatively associated with Ks, Kf, and Km in moderate and severe keratoconus eyes, and only a statistically significant association was found between SP-A1 and Ks in mild keratoconus eyes. It might be related to the definition of these keratometry parameters that Ks stands for the corneal dioptric power in the steepest meridian, Kf means the corneal dioptric power in the flattest meridian, and Km is the average value of Ks and Kf [37]. The cornea of keratoconus is characterized by localized thinning and forward bulging, and the discrepancy of the association between SP-A1 and different keratometry values might be related to the loss of structural integrity.

Corneal thickness is a basic parameter and could affect the biomechanical properties of the cornea [15]. It was reported that the thinning of the corneal apex is a typical character of the keratoconus eye [38]. The current results reported that SP-A1 in keratoconus eyes was statistically positively related with TCT, which was consistent with previous studies [16, 21, 32]. Several researchers had proposed that the thinning of the cornea in keratoconus patients would decrease the biomechanical properties, which in turn resulted in the focal weakening of cornea, and the decrease of corneal properties might further thin the cornea [15, 39, 40]. In addition, the study reported that SP-A1 in normal eyes was positively related to TCT, which is consistent with the findings of Heber et al. [32]. However, a recent study found no statistical correlation between SP-A1 and TCT in normal controls [16], which reminds us of the need to conduct a multicenter study to further investigate the correlation between SP-A1 and TCT, both in normal and keratoconus eyes.

The differences of SP-A1 in normal and keratoconus eyes indicated that SP-A1 could be used as an indicator to identify keratoconus eyes from normal eyes. Our further analyses showed that the SP-A1 had good diagnostic efficiency in detecting keratoconus eyes and mild keratoconus eyes from normal eyes, which was higher than our previous study [18] and the Kataria et al. [13] study, and slightly lower than the Sedaghat et al. [17] and Herber et al. study [32] findings. The definition of disease and the subject's character discrepancy in different studies might cause the results to be inconsistent, and a multicenter study should be conducted to confirm the diagnostic ability of SP-A1. SP-A1 is a resultant parameter measured by Corvis ST, and the relationship with other Corvis ST parameters in normal and keratoconus eyes is worthy of attention. SP-A1 was found to be statistically positively associated with IOP, bIOP, A1T, A2V, and negatively associated with DA, PD, A1V, A2T, and DA Ratio Max [2 mm], both in normal and keratoconus eyes, which is consistent with the study of Roberts et al. [3]. In addition, the influence of Radius, DA Ratio Max [1 mm], A1DLL, A2DLL, and HCDLL should be taken into account when using the SP-A1 in clinical application.

Although the study explored the association between SP-A1 and keratoconus severity indicators, the limitation should be noted. The subjects in the current study were almost from one center, which cannot directly be extrapolated to the rest of the population. However, the current results could, to some extent, represent the characteristic of SP-A1 in normal and keratoconus eyes, and further multicenter studies are warranted in the future.

In conclusion, the SP-A1 in keratoconus eyes was lower than in normal eyes, and was negatively associated with keratoconus severity. In addition, SP-A1 is helpful for clinical physicians in distinguishing mild keratoconus eyes from normal eyes. Further research should be conducted to explore the characteristics of SP-A1 and expand its clinical utility in other diseases.

## Figures and Tables

**Table 1:** Clinical parameters of normal and keratoconus eyes.

Parameters, median (IQR)	Normal eyes (*N* = 351)	Keratoconus eyes (*N* = 351)	Mild (*N* = 156)	Moderate (*N* = 142)	Severe (*N* = 53)	*P* ^*∗*^	*P* ^#^
Age (years)	24.00 (19.00, 27.00)	24.00 (20.00, 30.00)	24.00 (20.00, 29.50)	25.00 (20.00, 30.00)	23.00 (19.00, 29.00)	0.356	0.133
CDVA (LogMAR)	0.00 (0.00, 0.00)	0.22 (0.10, 0.40)	0.10 (0.00, 0.19)	0.30 (0.15, 0.40)	0.40 (0.30, 0.70)	<0.001	<0.001
IOP (mmHg)	15.50 (14.50, 17.00)	13.50 (11.88, 15.00)	13.50 (12.50, 15.00)	13.00 (11.00, 14.63)	12.50 (10.00, 14.00)	<0.001	<0.001
AL (mm)	25.42 (24.71, 26.21)	25.15 (24.35, 25.99)	25.25 (24.48, 26.07)	25.17 (24.34, 25.87)	24.42 (23.48, 25.78)	0.0104	0.311
ACD (mm)	3.73 (3.55, 3.91)	3.81 (3.68, 3.97)	3.78 (3.66, 3.90)	3.85 (3.71, 4.04)	3.87 (3.52, 4.14)	0.001	0.383
MCD (cells/mm^2^)	3001.00 (2814.00, 3199.00)	2766.00 (2576.00, 2989.50)	2772.00 (2617.50, 3040.50)	2784.00 (2564.50, 2961.00)	2643.00 (2502.00, 2782.00)	<0.001	<0.001
Ks (D)	43.89 (42.78, 44.70)	47.90 (45.80, 51.00)	45.80 (45.05, 47.10)	50.20 (49.00, 51.83)	57.60 (56.00, 61.40)	<0.001	<0.001
Kf (D)	42.66 (41.60, 43.40)	44.85 (43.70, 47.00)	43.90 (43.00, 44.65)	46.65 (44.90, 48.10)	51.70 (48.50, 56.20)	<0.001	<0.001
Km (D)	43.30 (42.20, 44.05)	46.35 (44.80, 49.13)	44.80 (44.08, 45.80)	48.20 (46.90, 49.83)	54.80 (52.40, 58.90)	<0.001	<0.001
TCT (*μ*m)	540.00 (519.00, 565.00)	461.50 (437.00, 492.00)	473.00 (446.50, 499.00)	458.00 (430.25, 478.25)	422.00 (389.00, 452.00)	<0.001	<0.001

CDVA, corrected distance visual acuity; IOP, intraocular pressure; AL, axial measurement; ACD, anterior chamber depth; MCD, mean endothelium cell density; Ks, steep keratometry; Kf, flat keratometry; Km, mean keratometry; TCT, thinnest corneal thickness. *P*^*∗*^ normal vs all keratoconus, *P*^#^ normal vs mild keratoconus after general linear model.

**Table 2:** Association between SP-A1 and Corvis ST parameters.

Parameters	Normal		Mild		Moderate		Severe	
	*r*	*P*	*r*	*P*	*r*	*P*	*r*	*P*
IOP	0.695	<0.001	0.659	<0.001	0.831	<0.001	0.807	<0.001
bIOP	0.397	<0.001	0.471	<0.001	0.704	<0.001	0.709	<0.001
DA	−0.631	<0.001	−0.654	<0.001	−0.719	<0.001	−0.678	<0.001
PD	−0.592	<0.001	−0.550	<0.001	−0.416	<0.001	−0.399	0.003
Radius	0.168	0.002	0.260	0.001	0.391	<0.001	0.264	0.056
A1T	0.546	<0.001	0.575	<0.001	−0.765	<0.001	0.744	<0.001
A1V	−0.746	<0.001	−0.655	<0.001	−0.530	<0.001	−0.545	<0.001
A1DLL	−0.107	0.045	−0.286	<0.001	−0.101	0.233	−0.059	0.683
A2T	−0.584	<0.001	−0.550	<0.001	−0.638	<0.001	−0.494	<0.001
A2V	0.542	<0.001	0.528	<0.001	0.523	<0.001	0.549	<0.001
A2DLL	−0.044	0.418	−0.151	0.062	−0.252	0.003	−0.137	0.347
HCT	0.021	0.701	0.001	0.989	0.055	0.518	0.223	0.108
HCDLL	−0.358	<0.001	−0.306	<0.001	−0.104	0.223	−0.084	0.564
DA Ratio Max [1 mm]	−0.474	<0.001	−0.376	<0.001	−0.509	<0.001	−0.199	0.152
DA Ratio Max [2 mm]	−0.620	<0.001	−0.594	<0.001	−0.681	<0.001	−0.463	<0.001

IOP, intraocular pressure; bIOP, biomechanical corrected intraocular pressure; DA, deformation amplitude; PD, peak distance; A1T, time from the initiation of air puff until the first applanation; A1V, corneal velocity at the first applanation; A1DLL, deflection length at the first applanation; A2T, time from the initiation of air puff until the second applanation; A2V, corneal velocity at the second applanation; A2DLL, deflection length at the second applanation; HCT, time from the initiation of air puff until the maximum deformation; HCDLL, deflection length at the maximum deformation.

## Data Availability

All relevant data are included within the article and its supporting information file and are available from the corresponding author upon request.

## Supplementary Materials

Supplementary Materials Supplementary Table 1: characteristics of other studies.

## References

[1] Mas Tur V., MacGregor C., Jayaswal R., O’Brart D., Maycock N. (2017). A review of keratoconus: diagnosis, pathophysiology, and genetics. *Survey of Ophthalmology*.

[2] Xu Z., Li W., Jiang J. (2017). Characteristic of entire corneal topography and tomography for the detection of sub-clinical keratoconus with Zernike polynomials using Pentacam. *Scientific Reports*.

[3] Roberts C. J., Mahmoud A. M., Bons J. P. (2017). Introduction of two novel stiffness parameters and interpretation of air puff-induced biomechanical deformation parameters with a dynamic Scheimpflug analyzer. *Journal of Refractive Surgery*.

[4] Vinciguerra R., Ambrósio R., Roberts C. J., Azzolini C., Vinciguerra P. (2017). Biomechanical characterization of subclinical keratoconus without topographic or tomographic abnormalities. *Journal of Refractive Surgery*.

[5] Mazzotta C., Baiocchi S., Bagaglia S. A., Fruschelli M., Meduri A., Rechichi M. (2017). Accelerated 15 mW pulsed-light crosslinking to treat progressive keratoconus: two-year clinical results. *Journal of Cataract & Refractive Surgery*.

[6] Rechichi M., Daya S., Scorcia V., Meduri A., Scorcia G. (2013). Epithelial-disruption collagen crosslinking for keratoconus: one-year results. *Journal of Cataract & Refractive Surgery*.

[7] Rechichi M., Mazzotta C., Daya S., Mencucci R., Lanza M., Meduri A. (2016). Intraoperative OCT pachymetry in patients undergoing dextran-free riboflavin UVA accelerated corneal collagen crosslinking. *Current Eye Research*.

[8] Raiskup F., Theuring A., Pillunat L. E., Spoerl E. (2015). Corneal collagen crosslinking with riboflavin and ultraviolet-a light in progressive keratoconus: ten-year results. *Journal of Cataract & Refractive Surgery*.

[9] Hashemi H., Ambrosio R., Vinciguerra R. (2019). Two-year changes in corneal stiffness parameters after accelerated corneal cross-linking. *Journal of Biomechanics*.

[10] Vinciguerra R., Ambrósio R., Elsheikh A. (2016). Detection of keratoconus with a new biomechanical index. *Journal of Refractive Surgery*.

[11] Mengyu W., Zhang Y., Wu W. (2018). Predicting refractive outcome of small incision lenticule extraction for myopia using corneal properties. *Translational Vision Science & Technology*.

[12] Fernández J., Rodríguez-Vallejo M., Martínez J., Tauste A., Salvestrini P., Piñero D. P. (2017). New parameters for evaluating corneal biomechanics and intraocular pressure after small-incision lenticule extraction by Scheimpflug-based dynamic tonometry. *Journal of Cataract & Refractive Surgery*.

[13] Kataria P., Padmanabhan P., Gopalakrishnan A., Padmanaban V., Mahadik S., Ambrósio R. (2019). Accuracy of Scheimpflug-derived corneal biomechanical and tomographic indices for detecting subclinical and mild keratectasia in a South Asian population. *Journal of Cataract & Refractive Surgery*.

[14] Vinciguerra R., Romano V., Arbabi E. M. (2017). In vivo early corneal biomechanical changes after corneal cross-linking in patients with progressive keratoconus. *Journal of Refractive Surgery*.

[15] Zhao Y., Shen Y., Yan Z. (2019). Relationship among corneal stiffness, thickness, and biomechanical parameters measured by Corvis ST, Pentacam and ORA in keratoconus. *Frontiers in Physiology*.

[16] Yaohua Z., Yan W., Liuyang L. (2018). Corneal stiffness and its relationship with other corneal biomechanical and nonbiomechanical parameters in myopic eyes of Chinese patients. *Cornea*.

[17] Sedaghat M.-R., Momeni-Moghaddam H., Ambrósio R. (2018). Diagnostic ability of corneal shape and biomechanical parameters for detecting frank keratoconus. *Cornea*.

[18] Yang K., Xu L., Fan Q. (2019). Repeatability and comparison of new Corvis ST parameters in normal and keratoconus eyes. *Scientific Reports*.

[19] Joda A. A., Shervin M. M. S., Kook D., Elsheikh A. (2016). Development and validation of a correction equation for Corvis tonometry. *Computer Methods in Biomechanics and Biomedical Engineering*.

[20] Qin X., Tian L., Zhang H. (2019). Evaluation of corneal elastic modulus based on corneal visualization Scheimpflug technology. *BioMedical Engineering OnLine*.

[21] Koh S., Inoue R., Ambrosio R. (2019). Correlation between corneal biomechanical indices and the severity of keratoconus. *Cornea*.

[22] Mercer R. N., Waring G. O., Roberts C. J. (2017). Comparison of corneal deformation parameters in keratoconic and normal eyes using a non-contact tonometer with a dynamic ultra-high-speed Scheimpflug camera. *Journal of Refractive Surgery*.

[23] Lee H., Roberts C. J., Ambrósio R., Elsheikh A., Kang D. S. Y., Kim T.-I. (2017). Effect of accelerated corneal crosslinking combined with transepithelial photorefractive keratectomy on dynamic corneal response parameters and biomechanically corrected intraocular pressure measured with a dynamic Scheimpflug analyzer in healthy myopic patients. *Journal of Cataract & Refractive Surgery*.

[24] Lee H., Roberts C. J., Kim T.-I., Ambrósio R., Elsheikh A., Kang D. S. Y. (2017). Changes in biomechanically corrected intraocular pressure and dynamic corneal response parameters before and after transepithelial photorefractive keratectomy and femtosecond laser-assisted laser in situ keratomileusis. *Journal of Cataract & Refractive Surgery*.

[25] Koc M., Aydemir E., Tekin K., Inanc M., Kosekahya P., Kiziltoprak H. (2019). Biomechanical analysis of subclinical keratoconus with normal topographic, topometric, and tomographic findings. *Journal of Refractive Surgery*.

[26] Herber R., Kaiser A., Grahlert X. (2020). Statistical analysis of correlated measurement data in ophthalmology: tutorial for the application of the linear mixed model in SPSS and R using corneal biomechanical parameters. *Ophthalmologe*.

[27] Vinciguerra R., Rehman S., Vallabh N. A. (2019). Corneal biomechanics and biomechanically corrected intraocular pressure in primary open-angle glaucoma, ocular hypertension and controls. *British Journal of Ophthalmology*.

[28] Ma J. N., Wang Y., Song Y. (2019). The effect of corneal biomechanical properties on opaque bubble layer in small incision lenticule extraction (SMILE). *Zhonghua Yan Ke Za Zhi*.

[29] Ma J., Wang Y. (2019). Comparative analysis of biomechanically corrected intraocular pressure with corneal visualization Scheimpflug technology versus conventional noncontact intraocular pressure. *International Ophthalmology*.

[30] Long W., Zhao Y., Hu Y. (2019). Characteristics of corneal biomechanics in Chinese preschool children with different refractive status. *Cornea*.

[31] Chan T. C. Y., Wang Y. M., Yu M., Jhanji V. (2018). Comparison of corneal tomography and a new combined tomographic biomechanical index in subclinical keratoconus. *Journal of Refractive Surgery*.

[32] Herber R., Ramm L., Spoerl E., Raiskup F., Pillunat L. E., Terai N. (2019). Assessment of corneal biomechanical parameters in healthy and keratoconic eyes using dynamic bidirectional applanation device and dynamic Scheimpflug analyzer. *Journal of Cataract & Refractive Surgery*.

[33] Sedaghat M.-R., Momeni-Moghaddam H., Ambrósio R. (2018). Long-term evaluation of corneal biomechanical properties after corneal cross-linking for keratoconus: a 4-year longitudinal study. *Journal of Refractive Surgery*.

[34] Riccardo V., Tzamalis A., Romano V., Arbabi E. M., Batterbury M., Kaye S. B. (2019). Assessment of the association between in vivo corneal biomechanical changes after corneal cross-linking and depth of demarcation line. *Journal of Refractive Surgery*.

[35] Shen Y., Han T., Jhanji V. (2019). Correlation between corneal topographic, densitometry, and biomechanical parameters in keratoconus eyes. *Translational Vision Science & Technology*.

[36] Zhang Y. Y., Jiang W. J., Teng Z. E. (2015). Corneal curvature radius and associated factors in Chinese children: the Shandong Children Eye Study. *PLoS One*.

[37] Penna R. R., de Sanctis U., Catalano M., Brusasco L., Grignolo F. M. (2017). Placido disk-based topography versus high-resolution rotating Scheimpflug camera for corneal power measurements in keratoconic and post-LASIK eyes: reliability and agreement. *International Journal of Ophthalmology*.

[38] Karimi A., Meimani N., Razaghi R., Rahmati S. M., Jadidi K., Rostami M. (2018). Biomechanics of the healthy and keratoconic corneas: a combination of the clinical data, finite element analysis, and artificial neural network. *Current Pharmaceutical Design*.

[39] Sinha Roy A., Dupps W. J. (2011). Patient-specific computational modeling of keratoconus progression and differential responses to collagen cross-linking. *Investigative Opthalmology & Visual Science*.

[40] Roberts C. J., Dupps W. J. (2014). Biomechanics of corneal ectasia and biomechanical treatments. *Journal of Cataract & Refractive Surgery*.

