# Supplementary material for: Association between Corneal Stiffness Parameter at the First Applanation and Keratoconus Severity
Source: J Ophthalmol. 2020 Dec 2;2020:6667507. doi: 10.1155/2020/6667507 (PMC7726963; doi:10.1155/2020/6667507)
Supplement: Supplementary Materials — Supplementary Table 1: characteristics of other studies. [file 6667507.f1.docx]

**Supplementary Table 1** Characteristics of other studies

| **NO** | **Author (year)** | **District** | **Eyes (subjects)** | **Age (years),**  **Mean±SD (range)** | **SP-A1 (mmHg/mm),**  **Mean±SD** | **IOP(mmHg),**  **Mean±SD** | **Pachy (µm), Mean±SD** | **K(D),**  **Mean±SD** | **Related parameters** |
| --- | --- | --- | --- | --- | --- | --- | --- | --- | --- |
| **Normal eyes** | |  |  |  |  |  |  |  |  |
| 28407167 | Cynthia J. Roberts(2017) | Brazil and Italy | 158(158) | 39.7±16.5 | 108.10±20.52 | bIOP: 14.2±2.0 | CCT: 542±34 |  | SP-A1 was significantly correlated with A1T, A1V, A2T, A2V, A2L, HC-radius, DA, PD, bIOP and CCT. |
| 28732615 | Joaquín Fernandez(2017) | Spain | 43(43) | 31.63±6.55  (22, 45) | 148.95±12.94 | IOP: 15.00±2.53  bIOP:14.68±1.99 |  |  | No statistically significant associations were found between SP-A1 and age. |
| 28880338 | Ryan N. Mercer(2017) | South Carolina | 47 |  | 96.4  (92.9, 103.2) |  |  |  |  |
| 28823441 | Hun Lee(2017) | South Korea | 69(69)  (Group 1: 35, Group 2: 34) | Group 1:  24.9±5.2  (19, 41)  Group 2:  25.3±5.5  (19, 40) | Group 1:  106.7±21.3,  Group 2:  98.1±18.9 | Group 1:  IOP: 16.93±2.17  bIOP:16.62±2.00  Group 2:  IOP: 17.10±2.10  bIOP:16.92±1.52 | Group 1:  CCT: 546.0±25.7  Group 2:  CCT: 543.2±28.5 | Group 1:  Km: 43.27±0.94  Group 2:  Km: 43.36±1.42 |  |
| 29335093 | Hun Lee(2017) | South Korea | 129(129) | 28.1±5.4  (20, 41) | 94.7±17.0 | IOP: 16.84±1.85  bIOP:16.21±1.66 | CCT: 559.9 ± 27.8 |  |  |
| 29847493 | Mohammad-Reza Sedaghat(2018) | Iran | 137(137) | 25.2±3.8  (19, 42) | 113.18±18.30 | IOP:16.33±1.68  bIOP:15.36±2.92 | CCT: 551.72±24.74  TCT: 547.48±24.39 | Km: 43.66±1.37 |  |
| 30527442 | Pratik Kataria(2018) | India | 100(100) | 24.00(20,37) | Median: 102.51  range:  (76.02, 131.86) | IOP:16.75(12.0, 27.5)  bIOP: 16.60(13.10, 24.00) | CCT:551.26±35.04 | Km:43.95(41.37, 48.85) | SP A1 was positivey related with IOP, bIOP and CCT. |
| 30199566 | Tommy C.Y. Chan（2018） | Hong Kong, China | 37(37) |  | 99.619 ± 16.191 |  | TCT: 561.5 ± 29.1 | Km: 42.92 ± 0.96 |  |
| 30271678 | Mengyu Wang(2018) | Tianjin, China | 151 (151) | 23.24±5.40 | 89.32±15.68 | IOP: 12.94±2.28  bIOP:13.55±1.94 | CCT: 553.28±26.8 |  |  |
| 29634670 | Yaohua Zhang(2018) | Tianjin, China | 387(387) | 23±5.53  (17, 44) | 90.46±15.39 | IOP:12.9±2.13  bIOP:13.5±1.85 | CCT: 554±25.78 | Km: 43.11±1.22 | The SP-A1 was significantly correlated with age, CCT, IOP, bIOP, Km, A1T, A1V, A2T, A2L, A2V, HC-radius and DA. No statistically significant associations were found between SP-A1, A1L, HCT and PD. |
| 30947733 | Xiao Qin(2019) | New Zealand | 20(10) | (20, 25) | 122.528±51.277 | IOP: 14.6±2.3 | CCT: 522±45 |  |  |
| 30984982 | Mustafa Koc(2019) | Turkey | 35(35) | 27.7±6.9  (16, 38) | 102.40±15.40 |  | TCT: 544.8±24.02 | K1: 42.27±1.37  K2: 44.14±1.04  Kmax:44.59±1.10 |  |
| 31478950 | Shizuka Koh(2019) | Japan | 70(70) | 42.8±13.8  (22, 80) | 110.24±16.60 |  | TCT: 544.90±9.86 |  |  |
| 31073680 | R. Herber(2019) | Deutschland | 158(79)  (Group 1: 29  Group 2: 27  Group 3: 23) | 45.6±16.8  (Group 1: 27.1±4.6  Group 2: 47.9±4.5  Group 3: 66.5±6.2) | Group 1:  99.8±2.32  Group 2:  102.0±2.40  Group 3:  112.4±2.66 | Group 1:  IOP: 14.3±0.33  bIOP: 13.8±0.28  Group 2:  IOP: 13.9±0.34  bIOP: 12.8±0.29  Group 3:  IOP: 15.0±0.37  bIOP: 13.0±0.31 | Group 1:  CCT: 564±4.51  TCT: 556.0±29.2(R)/  550.9±28.5(L)  Group 2:  CCT: 566± 4.68  TCT: 555.0±34.2(R)/ 549.1± 35.2(L)  Group 3:  CCT: 565±5.07  TCT:579.8±35.7(R)/ 545.0±35.9(L) | Kmax:  Group 1: 44.0±1.6(R)/ 44.1±1.5 (L)  Group 2: 44.3±1.8(R)/  44.5±1.8(L)  Group 3: 45.1±1.7(R)/  44.9±1.4(L) |  |
| 30902432 | R. Herber(2019) | Germany | 60(60) | 38.3 ± 12.8 | 103.09±17.72 | IOP:14.9 ±3.1 | TCT: 554±32 | Kmax: 44.3± 1.7 | SP-A1 was significantly affected by Age, Kmax, TCT and IOP. |
| 30923134 | Riccardo Vinciguerra(2019) | United Kingdom | 37(37) | 71.8±10.3 | 108.5±16.1 | IOP: 16.4±2.4  bIOP: 13.4±2.8 | CCT:553±33 |  |  |
| 30772990 | Jiaonan Ma(2019) | Tianjin, China | 129(71)  (Group 1: 31(20)  Group 2: 98(51)) | Group 1: 22.74±4.05  Group 2: 23.06±5.38 | Group 1:  108.85±13.77  Group 2:  100.19±14.13 |  | Group 1:  CCT: 572±29  Group 2:  CCT: 550±32 | Group 1:  K1: 42.64±1.23  K2: 43.90±1.30  Km: 43.25±1.21  Group 2:  K1: 42.83±1.27  K2: 44.20±1.55  Km: 43.50±1.36 |  |
| 31432352 | Jiaonan Ma(2019) | Tianjin, China | 1046(544) | 24.00±6.07  (18-48) | 101.315±14.228 | IOP:15.59±2.56 bIOP: 15.89±1.75 | CCT: 546±31.26 | K1:42.67±1.39,  K2: 44.03±1.55 | SP-A1 was associated with IOP and bIOP |
| 31033694 | Wen Long(2019) | Guangzhou, China | 128 (64)  (Group 1: 56, Group 2: 72) | Group 1:  5.57±0.60, Group 2: 5.07±0.86 | Group 1:  97.97±18.91  Group 2:  100.50±18.98 |  | Group 1:  CCT: 543.36±37.74  Group 2:  CCT: 551.97±24.66 | Group 1:  K1: 42.98±1.60  K2: 44.51±1.82  Group 2:  K1: 42.73±1.39  K2: 44.24±1.82 |  |
| 31263429 | Yu Zhao(2019) | Shanghai, China | 31 (31) | 25 | 93.68±21.93 |  | TCT: 540.58±21.93 | Kmax:44.42±1.59 | SP-A1 was significantly correlated with A1T, A1V, A2T, A2V, PD and DA. |
| 31653884 | KailiYang(2019) | Henan, China | 77(77) | 25.99±3.71 | 102.32±12.55 | IOP: 14.90±1.62  bIOP: 14.96±1.57 | CCT: 542.76±31.49 | K1: 42.73±1.23  K2: 43.68±1.37  Km: 43.21±1.27 |  |
|  | Current research | Henan, China | 351 | 24.11±5.38 | 112.61±16.22 | 15.56±2.63 | TCT: 552.96±62.35 | Km: 43.16±1.46 |  |
| **Keratoconus eyes** | |  |  |  |  |  |  |  |  |
| 28407167 | Cynthia J. Roberts(2017) | Brazil and Italy | 158(158) | 34.8±12.0 | 68.67±23.64 | bIOP: 14.4±1.9 | CCT: 471±36 |  | SP-A1 was significantly correlated with A1T, A1V, A1L, A2T, A2V, A2L, HC-radius, DA, PD, bIOP and CCT. |
| 28880338 | Ryan N. Mercer(2017) | South Carolina | 42 |  | 67.0  (61.4, 76.4) |  |  |  |  |
| 29227513 | Riccardo Vinciguerra(2017) | United Kingdom | 34(34) | 26±7 | 46.6±17.8 | bIOP: 13.3±1.73 | CCT: 467.2±33  TCT: 452.7±36.02 | Kmax: 56.3±8.32 |  |
| 29847493 | Mohammad-Reza Sedaghat(2018) | Iran | 145(145) | 24.9±6.0  (15, 47) | 60.91±22.93 | IOP: 14.00±1.27  bIOP:13.27±2.45 | CCT: 464.72±38.93  TCT: 455.66±38.04 | Km: 47.59±3.23 |  |
| 30540368 | Mohammad-Reza Sedaghat(2018) | Iran | 18(18) | 19.61±3.16  (15, 25) | 74.90±18.05 | IOP: 13.66±1.29 | CCT: 471.11±38.66 |  |  |
| 30527442 | Pratik Kataria(2018) | India | 100(100) | 23.50(12,42) | Median:77.16  range:(49.26, 147.60) | IOP:13.50(7.0, 21.0)  bIOP: 15.20(8.60, 20.20) | CCT:497.18±31.87 |  |  |
| 31478950 | Shizuka Koh(2019) | Japan | 53(53) | 38.5±12.2  (12, 70) | 67.22±22.28 |  | TCT: 432.90±69.3 |  | SP-A1 was significantly correlated with TCT. |
| 30984982 | Mustafa Koc(2019) | Turkey | 21(21) | 26.1±5.8  (16, 36) | 67.40±25.38 |  | TCT: 468.2±67.00 | K1: 43.59±4.03  K2: 47.00±4.04  Kmax:50.38±5.84 |  |
| 31300155 | Hassan Hashemi(2019) | Tehran | 37  (Group 1: 16  Group 2: 27) | Group 1:  21.7 ± 4.9  Group 2:  21.5 ± 5.2 | Group 1:  73.19±19.31  Group 2:  61.91±14.22 | Group 1:  bIOP: 14.87±2.2  Group 2: bIOP:15.09±1.92 | TCT≥400.0 | Kmax < 55.0 D |  |
| 30855098 | Riccardo Vinciguerra (2019) | United Kingdom | 66(66) | 24 ± 6 | 60.3 ± 18.8 | bIOP:13.3 ± 2.34 | TCT：489.8±34.67 | Kmax:56.65 ±7.91 |  |
| 30902432 | R. Herber(2019) | Germany | 60(60) | 38.3±12.8 | 61.36± 17.28 | IOP: 13.9 ± 3.3 | TCT: 459 ± 47 | Kmax: 55.4 ± 6.3 | SP-A1 was significantly affected by Kmax, TCT and IOP. |
| 31110913 | Yang Shen(2019) | Shanghai, China | 76(76) | 23.93±6.81 | 61.14±20.21 | IOP: 14.11±2.68  bIOP:15.29±2.63 | CCT: 495.3±37.7  TCT: 477.0±44.0 | K1: 46.63±5.06  K2: 49.98±6.08  Km: 48.15±5.32  Kmax:56.25±9.41 |  |
| 31263429 | Yu Zhao (2019) | Shanghai, China | 75 (44)  (mild:31, moderate:27, severe:17) | 22 | 61.6 ±21.87 |  | TCT: 453.64±81.53 | Kmax:  57.62±10.76 | SP-A1 was significantly correlated with A1T, A1V, A2T, PD, R, and DA in mild keratoconus eyes (Kmax<55D), and correlated with A1T, PD and DA in moderate keratoconus eyes (55 D≤Kmax<62D), and correlated with A1T, A1V, A2V, PD and DA in severe keratoconus eyes (Kmax≥62D). |
| 31653884 | KailiYang(2019) | Henan, China | 77(47) | 24.58±5.19 | 69.90±22.44 | IOP: 13.26±2.53  bIOP: 14.64±2.36 | CCT: 485.77±45.09 | K1: 46.69±4.46  K2: 49.37±5.36  Km: 47.65±6.13 |  |
|  | Current research | Henan, China | 351 | 24.82±6.75 | 70.87±19.94 | 12.49±2.80 | TCT: 457.59±42.16 | Km: 57.62±10.76 |  |

IOP, intraocular pressure; bIOP, biomechanical corrected intraocular pressure; K1, flatest keratometry (Kf); K2, the steepest keratometry; Km, mean keratometry; Kmax, the max keratometry; SP-A1, stiffness parameter at first applanation; CCT, central corneal thickness; TCT, thinnest corneal thickness.
